# Effects of Pimobendan on Prolonging Time to Rehospitalization or Frequency of Rehospitalization in Patients with Heart Failure: A Retrospective Cohort Study Using a Medical Administrative Database (PREFER Study)

**DOI:** 10.36469/jheor.2020.13246

**Published:** 2020-06-23

**Authors:** Kazuhisa Kodama, Tatsunori Murata, Naoki Dohi, Masaaki Nakano, Toshiaki Yokoi, Tomohiro Sakamoto, Koichi Nakao

**Affiliations:** 1 Division of Cardiology, Saiseikai Kumamoto Hospital Cardiovascular Center, Kumamoto, Japan; 2 Crecon Medical Assessment Inc., Tokyo, Japan; 3 Medical Affairs, Toa Eiyo Ltd., Tokyo, Japan

**Keywords:** *pimobendan*, heart failure, propensity score matching method, phosphodiesterase III inhibitor, calcium sensitizer, oral inotropic agent, epidemiology

## Abstract

**Background:**

As approximately 24% of patients with chronic heart failure are rehospitalized within 1 year and heart failure is aggravated by repeated hospitalizations, greater importance was attached to the prevention of hospitalization.

**Objective:**

The objective of this study was to investigate the influence of pimobendan on rehospitalization of patients with advanced heart failure using a Japanese medical administrative database.

**Methods:**

From January 2010 to February 2018, patients hospitalized two or more times for heart failure were selected for analysis. The primary endpoint was the incidence of hospitalizations for heart failure during the follow-up period, which was compared between pimobendan prescription and non-prescription groups after propensity score matching.

**Results:**

The total number of patients with heart failure included during the study period was 1 421 110 and we matched 276 patients in both groups. The incidence of rehospitalization throughout the period to completion of follow-up was 365.23/1000 people/yr (95% confidence interval [CI]: 327.78–402.69) in the pimobendan prescription group and 537.81/1000 people/yr (95% CI: 492.36–583.27) in the non-prescription group. The cumulative incidence at 365 days was significantly lower in the pimobendan prescription group (pimobendan prescription group: 35.4% (95% CI: 29.8–41.8), non-prescription group: 51.2% (95% CI: 45.1–57.7), (*P* < 0.001). The adjusted hazard ratio in the pimobendan prescription group was 0.556 (95% CI: 0.426–0.725, *P* < 0.001).

**Conclusion:**

Pimobendan was suggested to extend the time to rehospitalization for patients with advanced heart failure. It is necessary to verify the results of this study by performing a prospective study. In addition, the influence of pimobendan on general heart failure patients must be examined.

## INTRODUCTION

The 5-year survival rate for heart failure is only 50%, and approximately 24% of patients with chronic heart failure are rehospitalized within 1 year.^1^,^2^

In the Japanese Cardiac Registry of Heart Failure in Cardiology trial, a large-scale, investigative study involving patients with aggravated chronic heart failure requiring inpatient treatment, the majority (73%) of all patients were 65 or more years old and the mean age was 71,^3^ confirming that many heart failure patients are elderly. However, conventional heart failure practice guidelines were developed based on studies conducted on relatively young patients although the majority of heart failure treatment patients are elderly. The Japanese Heart Failure Society announced in 2016 their Statement on treatment of elderly heart failure patients,^4^ in which maintenance and improvement of quality of life (QOL) for elderly patients with heart failure were positioned as major treatment objectives. Greater importance was attached to the prevention of hospitalization because heart failure is aggravated by repeated hospitalizations.

Pimobendan is an oral calcium sensitizer and phosphodiesterase (PDE) III inhibitor that has been approved for use only in Japan. While, no significant cardiovascular improvements were noted for pimobendan in past clinical studies (PICO trial and EPOCH trial), improvements in exercise tolerance and QOL were confirmed.^5^,^6^

An observational study performed recently in Japan suggested that admission for heart failure can be prevented by an oral inotropic agent administered to patients with chronic heart failure;^7^ however, epidemiological studies on pimobendan are limited.

The objective of this study was to investigate the relationship between pimobendan usage and rehospitalization in patients with advanced heart failure using a Japanese medical administrative database. In addition, adverse events related to pimobendan were also examined.

## METHODS

### Study Design

This was a retrospective observational study conducted using the propensity score matching method. Data was obtained from a medical administrative database, EBM Provider^®^ provided by Medical Data Vision Co., Ltd. (MDV).

The EBM Provider database covers approximately 18% of all Japanese acute-phase medical care institutions that have implemented the diagnosis procedure combination (DPC) system. EBM Provider is constructed based on DPC data and claims data for which the hospitals have approved secondary use. All data are anonymized for the protection of personal information. Studies using EBM Provider have been described previously (including one report by Nakao et al.).^8^ As EBM Provider is a pooled database containing data from individual medical institutions, treatment information for patients prior to admission was only available when patients were treated in the same hospital. Also, patients cannot be followed after transfer to another hospital.

Data sets for analysis were provided directly to Crecon Medical Assessment Inc. from MDV. Other than Murata, the authors received only the analysis results and did not receive the analysis data set.

### Ethical Approval

The protocol for this study was approved by the ethical review board of Saiseikai Kumamoto Hospital. This study was registered to the University Hospital Medical Information Network (UMIN; https://www.umin.ac.jp/ctr/index.htm; UMIN000034228).

### Patient Selection

The study period was January 2010 to February 2018. To investigate the relationship between pimobendan usage and rehospitalization in patients previously hospitalized for heart failure, those meeting the following inclusion criteria were selected for analysis: (1) patients hospitalized two or more times during the study period for heart failure, (2) patients that had two or more outpatient visits after the next day of discharge from the second hospitalization for heart failure (i.e., the index date of follow-up start), and (3) patients 20 or more years old at the index date.

Patients meeting the following criteria were excluded: (1) Japan Coma Scale score of 1 or more upon discharge from the index hospitalization (i.e., the second hospitalization for heart failure), (2) activities of daily living (ADL) score of 12 or more upon discharge from the index hospitalization, (3) short-term prescription with a percentage of pimobendan prescription days in follow-up duration of less than 80% in the pimobendan prescription group, (4) prescription of vesnarinone, denopamine, or docarpamine during follow-up, (5) prescription of inotropic agent injections (dopamine hydrochloride, dobutamine, PDE III inhibitor, or colforsin daropate) during follow-up, (6) treatment by cardiac surgery (coronary artery bypass, valve replacement, valvuloplasty, etc.), transcatheter aortic valve implantation (TAVI), cardiac resynchronization therapy (CRT), or percutaneous coronary intervention (PCI) within 90 days before the index date, and (7) diagnosis or treatment of cancer, heart transplantation, or placement of a ventricular assist device during the study period. In addition, patients with a missing covariate at the time of calculating the propensity score were excluded.

The patient population prescribed pimobendan during the index hospitalization or during follow-up was designated as the pimobendan prescription group. Patients that did not receive pimobendan were assigned to the non-prescription group.

Consecutive hospitalizations were regarded as a series when “leading diagnosis to admission” (required input in DPC systems) was the same in two consecutive hospitalizations and the time of discharge from the first hospitalization was within 7 days before the second admission.

## STUDY MEASURE

### Patient Characteristics

Patient demographics included age, sex, body mass index, smoking status, year that follow-up began, and the interval between hospital admissions for heart failure. Clinical patient characteristics included ADL score, cardiac function classification of the New York Heart Association (NYHA), Charlson comorbidity index, underlying heart diseases (ischemic heart disease, cardiomyopathy, valvular disease, arrhythmia, and other heart diseases), information on the baseline hospital visit occurring before the follow-up start point (inpatient clinical department, discharged from hospital, ambulance transport, duration [days] spent in hospital, and duration [days] spent in an intensive care unit), concomitant drugs and therapy (inotropic agents [intravenous injection], diuretics [intravenous injection], tolvaptan, carperitide, intra-aortic balloon pumping or percutaneous cardio pulmonary support, extracorporeal membrane oxygenation, artificial respiration or nasal mask-type support ventilation, right heart catheter, left heart catheter, bilateral heart catheter, or blood transfusion) during baseline hospitalization at the follow-up start point, concomitant drugs and concomitant therapy (angiotensin-converting–enzyme inhibitor [ACE-I] or angiotensin II receptor blocker [ARB], β blocker, digitalis preparation, diuretics [excluding tolvaptan and mineralocorticoid receptor antagonist], tolvaptan, mineralocorticoid receptor antagonist, Ca antagonist, statin, nitrate, aspirin, warfarin, direct oral anticoagulants, cardiac rehabilitation, implantation of cardiac resynchronization therapy pacemaker [CRT-P] or cardiac resynchronization therapy defibrillator [CRT-D], implantation of implantable cardioverter defibrillator [ICD], home oxygen therapy, and adaptive support ventilation) during follow-up, and concomitant diseases (atrial fibrillation, cerebrovascular disease, chronic obstructive pulmonary disease, diabetes, including abnormal glucose tolerance, hypertension, dyslipidemia, renal disease, liver disease, hyperuricemia, anemia, and dialysis) and surgery for heart disease (PCI, excimer laser, pacemaker implantation, external pace making, coronary artery bypass grafting, surgical left ventricular restoration, closure of ventricular septal perforation or repair of left ventricular free wall rupture, percutaneous transluminal myocardial ablation, valvuloplasty, and valve replacement), during the baseline hospitalization, and at the follow-up start point, and during follow-up. In addition, the duration [months] of follow-up at the time of primary endpoint analysis and frequency of visits during follow-up were also included in the patient background information.

### Outcomes

The primary endpoint was the incidence of hospitalizations for heart failure during the follow-up period, which was compared between pimobendan prescription and non-prescription groups. Secondary outcome measures included the annual frequency of hospitalizations for heart failure and all-cause hospitalizations during follow-up, which were also compared between both groups. Furthermore, the incidence of hospitalization for adverse events related to pimobendan occurring in the follow-up period was investigated for torsade de pointes, transient ventricular fibrillation, ventricular fibrillation, ventricular tachycardia, nonsustained ventricular tachycardia, and pulseless ventricular tachycardia. The ICD-10 codes and disease codes in Japan for these endpoints are as follows: heart failure (ICD-10: I11.0, I50), torsade de pointes (ICD-10: I472, I490, disease code: 8847804), transient ventricular fibrillation (ICD-10: I490, disease code: 4274001), ventricular fibrillation (ICD-10: I490, disease code: 4274004), ventricular tachycardia (ICD-10: I472, disease code: 4271005), nonsustained ventricular tachycardia (ICD-10: I472, disease code: 8847767), and pulseless ventricular tachycardia (ICD-10: I472, disease code: 8847822).

### Follow-Up Definition

**Figure 1:**
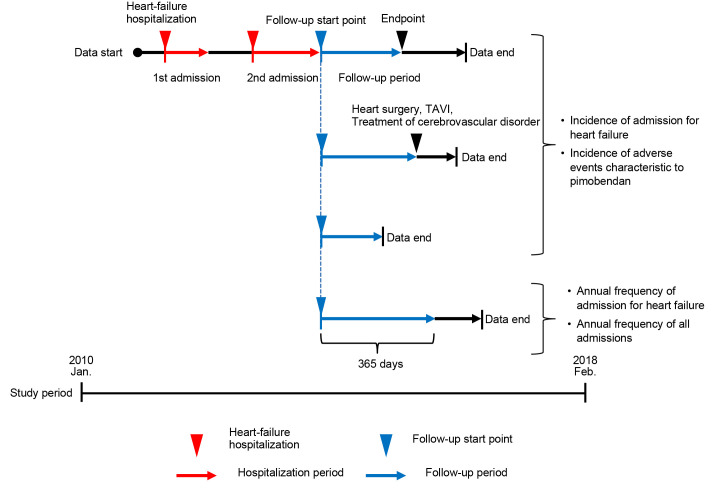
Follow-Up Start Point and Follow-Up Period by Outcome

The follow-up period for endpoints on hospitalization for heart failure and adverse events characteristic to pimobendan ended when the following three conditions were observed: (1) development of the endpoint, (2) execution of cardiac surgery (coronary artery bypass, valve replacement, valvuloplasty, etc.), TAVI, or treatment of cerebrovascular disorder, and (3) the final recorded day on EBM Provider (the last day of the month). The follow-up period for the annual frequency of admission for heart failure and annual frequency of all-cause hospitalization was 365 days from the index date (patients who were unable to be followed for 365 days or longer were excluded from the analysis set) (Figure 1).

### Statistical Analysis

Statistical analysis was performed by Crecon Medical Assessment Inc. Matching analysis employing the propensity score method was performed to adjust the patient background and increase comparability between the pimobendan prescription and non-prescription groups. The probability of allocation of each patient to the pimobendan prescription group (propensity score) was calculated using logistic regression analysis. The items included as the patient background were considered as covariates for calculation of the propensity score, and the following items strongly correlated with other covariates exhibiting multicollinearity were excluded: concomitant drugs and therapy (extracorporeal membrane oxygenation, right heart catheter, left heart catheter) during baseline hospitalization at the follow-up start point, concomitant drugs and concomitant therapy (statin, implantation of ICD) during follow-up, and concomitant diseases (atrial fibrillation) during the baseline hospitalization at the follow-up start point and during follow-up, the duration [months] of follow-up at the time of primary endpoint analysis, and frequency of visits during follow-up. One-to-one nearest neighbor matching within the caliper of width equal to 0.2 of the standard deviation of the logit of the propensity score without replacement was used.^9^ A between-group balance check was performed using the standardized mean difference.

For time-to-event data, the incidence in 1000 persons per year and its 95% CI were calculated. The chi-square test was used for comparisons between groups. Regarding rehospitalization for heart failure, the cumulative incidence was estimated using the Kaplan–Meier method. The cumulative incidence at 365 days after the initiation of follow-up and 95% CI were calculated. The log-rank test was used for between-group comparison. In addition, the hazard ratio and its 95% CI were calculated using the Cox proportional hazard model. For the populations matched using the propensity score method, the hazard ratio was calculated using the Cox proportional hazard model with robust variance estimator regarding the propensity score as a covariate.

For continuous value data, the mean, standard deviation (SD), minimum value, median, and maximum value were presented. For between-group comparison, the t test was used for the patient background items, whereas the Wilcoxon signed-rank test was used for the interval between hospitalizations for heart failure, annual frequency of rehospitalization for heart failure, and annual frequency of all-cause hospitalizations.

For categorical data, the number of cases and its rate among all cases were presented. The chi-square test was used for between-group comparison.

All analyses were performed using SAS version 9.4 (SAS Institute Inc., Cary, NC).

### Sensitivity Analysis

Using the propensity score calculated for matching, the sensitivity analysis using the inverse probability of treatment weighting (IPTW) method were also performed to test a potential bias due to a sample exclusion by propensity score matching process.

In addition, the sensitivity analysis for the patients stratified into populations based on the interval between the first and second index hospitalizations served as the baseline of the follow-up start point: shorter than 24 months, shorter than 12 months, shorter than 6 months, and shorter than 3 months, were also performed.

## RESULTS

### Study Population

**Figure 2:**
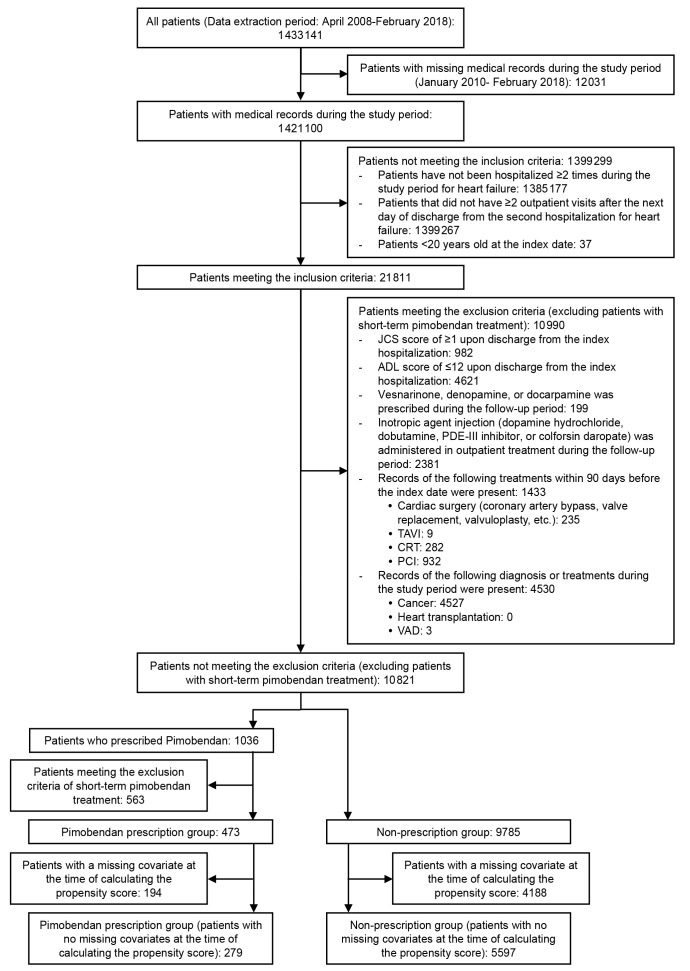
Patient Flow Chart for Evaluating the Incidence of Rehospitalization for Heart Failure Abbreviations: ADL, activities of daily living; PDE, phosphodiesterase; TAVI, transcatheter aortic valve implantation; CRT, cardiac resynchronization therapy; PCI, percutaneous coronary intervention; VAD, ventricular assist device.

The total number of patients with heart failure included during the study period was 1 421 110 and finally 5876 patients were enrolled for evaluation of the primary endpoint (pimobendan prescription group: 279, non-prescription group: 5597). A patient flow chart for the primary endpoint (incidence of rehospitalization for heart failure) is shown in Figure 2. The number of patients enrolled for evaluation of the annual frequency of hospitalization for heart failure or annual frequency of allcause hospitalizations was 4090 (pimobendan prescription group: 254, non-prescription group: 3836) and the number of patients enrolled for evaluation of the incidence of hospitalization for adverse events characteristic to pimobendan was 5032 (pimobendan prescription group: 252, non-prescription group: 4780). The number of patients in each treatment group differed for each endpoint due to variations in the length of the follow-up period.

### Patient Characteristics

Table 1 shows the number of patients in each matched group as well as selected background information.

The mean age after propensity score matching was approximately 74 years old. The mean ADL score at discharge was high (i.e., relatively good) at 19.4 in both prescription and non-prescription groups. The percentage of patients discharged to home was also high (over 96%) in both groups. Cardiac rehabilitation was performed for 45 patients during follow-up (pimobendan prescription group: 24, nonprescription group: 21) and during hospitalization for approximately 50% (pimobendan prescription group: 11, non-prescription group: 13).

For the primary endpoint, the mean follow-up duration was 16.7 months in the pimobendan prescription group and 12.7 months in the non-prescription group (*P* = 0.001). The mean frequency of visits during follow-up was 1.9 days/month in the pimobendan prescription group and 1.6 days/month in the non-prescription group (*P* = 0.059), with no significant difference between the groups (Table 2).

The standardized mean difference of the selected patient background items mostly ranged from -0.1 to 0.1 (Table 3). The largest deviation in the standardized mean difference from 0 was noted in the presence or absence of concomitant statin treatment during follow-up (0.18). The standardized mean differences of the other items also mostly ranged from −0.1 to 0.1.

The mean daily dose of pimobendan in the pimobendan prescription group during follow-up was 3.1 mg (SD: 1.4, first quartile: 2.5, median: 2.5, third quartile: 5.0). In addition, the hospital category (university, public, private, etc.) and the number of hospital beds were not significantly different between the groups (data not shown).

### Incidence of Rehospitalization for Heart Failure

The incidence of rehospitalization throughout the period to completion of follow-up was 365.23/1000 people/yr (95% CI: 327.78–402.69) in the pimobendan prescription group and 537.81/1000 people/yr (95% CI: 492.36–583.27) in the non-prescription group (Table 4).

**Figure 3:**
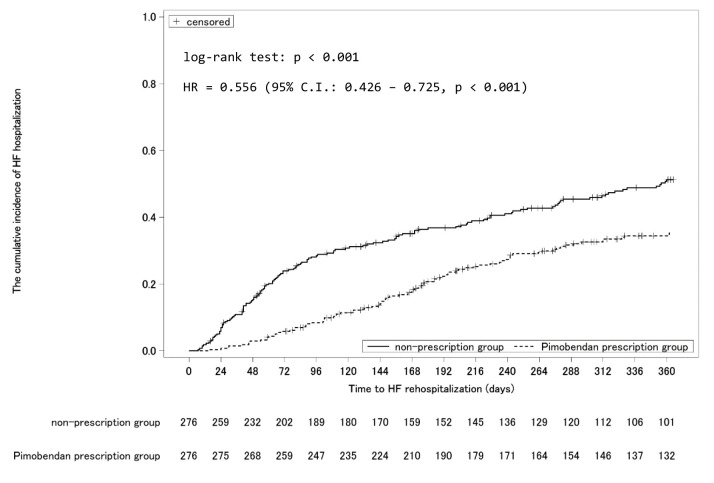
Cumulative Incidence of Rehospitalization for Heart Failure During the 1-year Period Following Initiation of Follow-Up Abbreviation: HF, heart failure. The *P* value and hazard ratio of the log-rank test were determined using the data of the 1-year period after initiation of follow-up.

**Figure 4:**
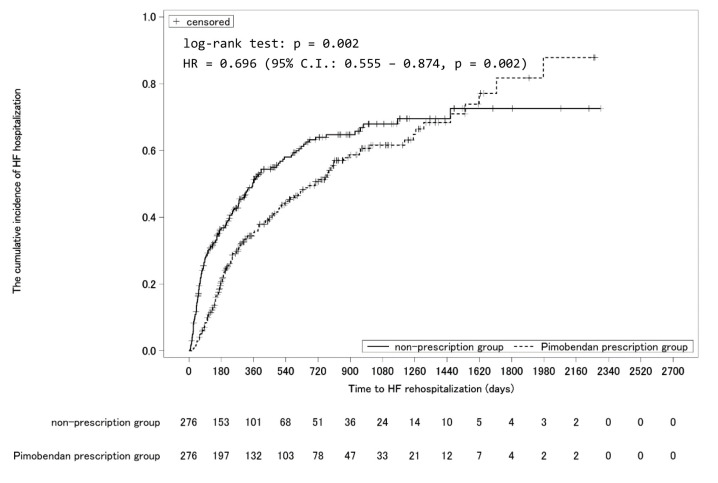
Cumulative Incidence of Rehospitalization for Heart Failure Throughout the Period to Completion of Follow-Up Abbreviation: HF, heart failure.

The cumulative incidence from initiation of follow-up to 365 days was estimated using the Kaplan–Meier method (Figure 3). The cumulative incidence at 365 days was significantly lower in the pimobendan prescription group (pimobendan prescription group: 35.4% (95% CI: 29.8–41.8%), non-prescription group: 51.2% (95% CI: 45.1–57.7%), *P* < 0.001). The adjusted hazard ratio in the pimobendan prescription group was 0.556 (95% CI: 0.426–0.725, *P* < 0.001). The cumulative incidence until the completion of follow-up is shown in Figure 4. The cumulative incidence-decreasing effects of pimobendan were lost at approximately 1500 days from the initiation of follow-up.

### Annual Frequency of Rehospitalization for Heart Failure and Annual Frequency of All-Cause Hospitalizations

The mean annual frequency of rehospitalization for heart failure was significantly lower in the pimobendan prescription group (pimobendan group: 0.5, non-prescription group: 0.8, *P* = 0.021) (Table 5). There is no significant difference in the mean annual frequency of all-cause hospitalizations between the groups (pimobendan group: 1.0, non-prescription group: 1.2, *P* = 0.133).

### Incidence of Adverse Events

The incidence of adverse events characteristic to pimobendan is shown in Table 6. The number of cases was small in both groups.

### Sensitivity Analysis

In order to confirm the robustness of the results in the matched population since a lot of patients were excluded from non-prescription group by matching process, sensitivity analysis using the IPTW method was performed. The results were almost consistent with those of the matching analysis.

**Figure 5:**
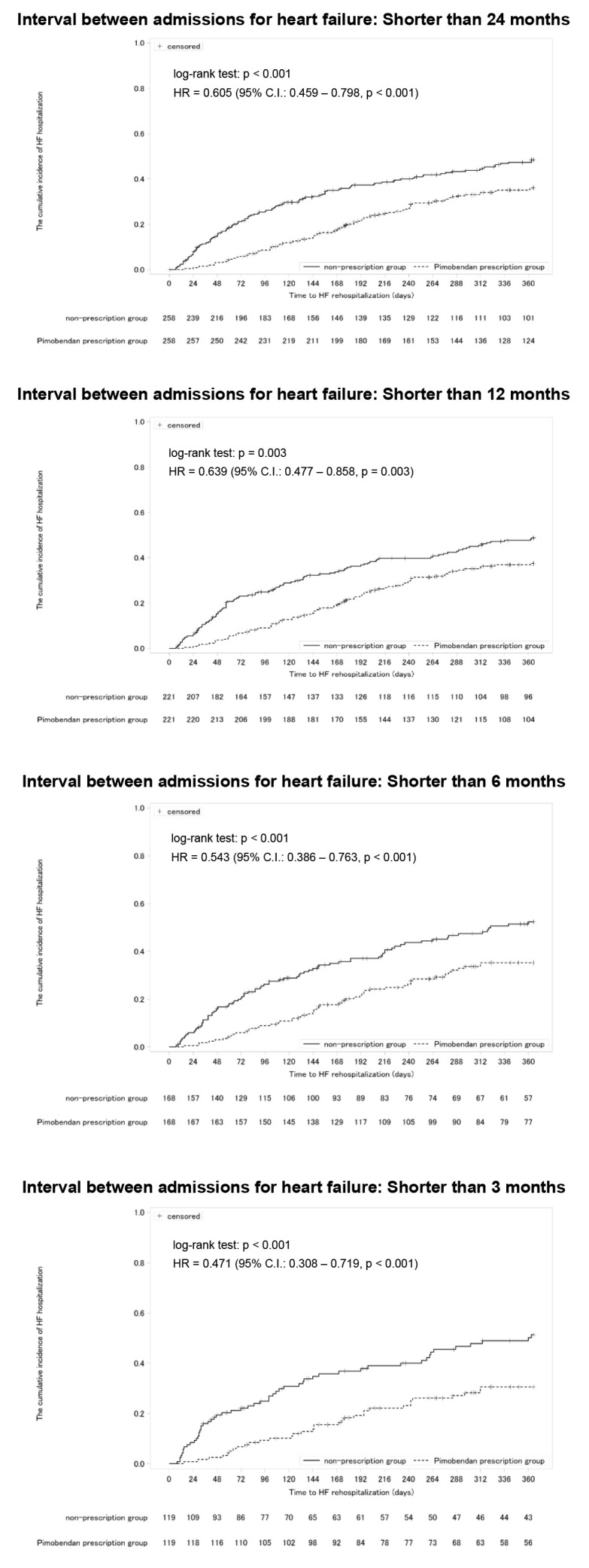
Cumulative Incidences of Rehospitalization for Heart Failure Stratified Based on the Interval of Index Hospitalizations Abbreviation: HF, heart failure. Cumulative incidences of rehospitalization for heart failure at the follow-up start point of patient populations with an interval between the first and second hospitalizations for heart failure, which served as the baseline of the follow-up start point, shorter than 24 months, shorter than 12 months, shorter than 6 months, or shorter than 3 months in the 1-year period after the initiation of follow-up. The *P* value and hazard ratio of the log-rank test were determined using the data of the 1-year period after the initiation of follow-up.

The results of the sensitivity analysis limiting the interval between the first and second index hospitalizations for heart failure were also consistent with those of the basic analysis (Table 7, Figure 5).

## DISCUSSION

We investigated the relationship between pimobendan and rehospitalization in patients with heart failure. A propensity score method was used to create matched groups. Pimobendan treatment reduced the incidence and cumulative incidence of rehospitalization for patients with advanced heart failure who were hospitalized repeatedly. The number of hospitalizations for adverse events related to pimobendan treatment was low and insufficient for confirmatory discussion in both groups.

The cumulative incidence of rehospitalization estimated using the Kaplan–Meier method was lower in the pimobendan prescription group at 365 days, but the difference between the groups disappeared at approximately 1500 days. As follow-up would have already been complete for many patients, this may have been due to the small number (about 10) of patients available for analysis at this time, reducing the comparability of the groups. Thus, this finding should be very carefully interpreted.

The rate of discharge to home (97.3%) and mean ADL score at discharge (19.4, representing high independence) was high for all matched patients, which suggested patient cognitive function was favorable and that drugs could be taken by patients themselves, perhaps because of the relatively low mean age of 74. For comparison, the median age in the latest epidemiological study using the Kyoto Congestive Heart Failure registry by Yaku et al. was 80 years old.^10^ In addition, the frequency of inotropic agent injections during hospitalization was higher than that in the acute decompensated heart failure syndromes registry.^11^ The rate of tolvaptan used during hospitalization as the baseline of the follow-up period was also higher,^8^ suggesting that many relatively advanced patients were included.

A decrease in patients with a follow-up start year of 2016 or later was noted. This may have been due to an increase in patients missing NYHA records at discharge (necessary to calculate the propensity score); however, they became unessential for DPC input requirements in April 2016.

Patient characteristics were balanced between matched groups, but the standardized mean difference of statins was relatively large at 0.18. While the rate of statin use was higher in the pimobendan prescription group before matching, it was higher in the nonprescription group after matching. The relationship of statins has been reported to be beneficial for patients with heart failure in observational and small-scale clinical studies, but such effects were not observed in large-scale clinical studies.^12^,^13^ As there has been no specific consensus in preceding studies, it is unclear how statins affected the results of the present study.

The sensitivity analysis in patients stratified based on the interval between the first and second index hospitalizations for heart failure: shorter than 24 months, 12 months, 6 months, and 3 months, was also performed. The incidence and cumulative incidence of rehospitalization for heart failure in each sensitivity analysis were consistent with those in the entire population. Based on these findings, pimobendan is expected to extend the time to rehospitalization for heart failure to the same degree for advanced patients with a high risk of rehospitalization for heart failure.

Prospective intervention studies (PICO, EPOCH, etc.) conducted in the time period for which β-blockers had been inactively administered did not show a prognostic improvement effect, but the improvement of QOL was known.^5^,^6^ On the other hand, in a recent report from an observational study of a small number of cases, a decrease in the number of hospitalizations for heart failure after administration of pimobendan was reported.^7^ The reason for extending the interval to hospitalization for heart failure is unclear, but the improvement of QOL due to the inotropic and vasodilatory effects by pimobendan may suggest suppression of heart failure hospitalization. In addition, administration of cardioprotective drugs such as β-blockers suppresses fatal arrhythmia events, and pimobendan is considered to extend the interval by administering the drug safely inasmuch as the cardioprotective drugs come into effect.

In addition, the slope of the Kaplan–Meier curve immediately after the follow-up initiation was steeper in the non-prescription group than that in the pimobendan prescription group. Similarly, Silvetti et al. reported that levosimendan, an intravenous calcium sensitizer and PDE III inhibitor, was associated with a reduction in the rehospitalization rate at 3 months in patients with acute advanced heart failure.^14^ The results from Silvetti et al. are consistent with and supportive of our results. These suggest that pimobendan would also contribute to prolonging the time to rehospitalization in Japanese patients with advanced heart failure, especially in the vulnerable postdischarge phase.

This study suggests that pimobendan has potential to reduce the incidence of rehospitalization for heart failure patients with repeated hospitalization. However, pimobendan is not necessarily recommended for all such heart failure patients. Administering pimobendan at a low dose to patients for whom the drug is expected to be effective (based on the pathology after treatment following the heart failure guidelines and statements), we interpreted that pimobendan can be expected to contribute in managing repeated hospitalization and discharge thus, improving patients’ QOL.

## STUDY LIMITATIONS

There are several limitations in this study that have been raised because this analysis was based on a medical administrative database that contains data collected from hospitals in Japan where DPC systems were introduced.

The first limitation is the internal validity of this study. The patient backgrounds of the pimobendan prescription and non-prescription groups were adjusted by matching analysis using the propensity score to increase comparability between the groups. Factors considered in the calculation of the propensity score were acquired from DPC data and claims data in EBM Provider. Unmeasurable factors, such as renal function, nutritional state, and social factors, were not adjusted for. Moreover, there are instances where the same patient was handled under different identifiers at different medical institutions in EBM Provider. Thus, information before hospitalization is available only for patients who visited the same hospital throughout.

The second limitation is generalizability. Short-term prescription patients were excluded from the pimobendan prescription group. As a result, the proportion of responders to pimobendan probably increased and the effects of pimobendan on rehospitalization risk for heart failure may have been overestimated. In addition, patients included in the analysis set were limited to those who were hospitalized repeatedly with advanced heart failure, thus applicability to patients with milder general heart failure is unclear.

The third point is related to the accuracy of patient follow-up. If the patient was admitted to another medical institution for heart failure during follow-up, they were unable to be evaluated, leading to underestimation of incidence and annual frequency of rehospitalization. However, although the frequency of visits during follow-up may have decreased in such cases, no major difference was noted in the frequency of visits during follow-up between both groups. Thus, the impact of this limitation on the conclusion may be limited.

The fourth limitation is the matching analysis using the propensity score method. As the number of patients was more than 20 times larger in the non-prescription group before matching, many patients in the non-prescription group were excluded by matching, which may have generated a bias in the results. However, when sensitivity analysis was performed using the IPTW method, the results were consistent with those on matching, suggesting that the impact of this limitation on the study’s conclusion was also small.

The fifth limitation is the impact on life prognosis. Since this study did not assess mortality because the data source used in the study have limited information for death (i.e., death information only during hospitalization is available), the effects of pimobendan prescription on life prognosis cannot be discussed from the results of this study.

## CONCLUSION

Pimobendan was suggested to extend the time to rehospitalization for patients with advanced heart failure with resulting rehospitalization. Attempts have been made to increase comparability using the propensity score method; however, this result should not be practiced directly and some important limitations remain. It is necessary to verify the results of this study by performing a prospective study.

## Figures and Tables

**Table 1:** Patient Characteristics for Evaluation of the Incidence of Rehospitalization for Heart Failure^a^

		Before Propensity Score Matching							After Propensity Score Matching						
		All		Pimobendan Prescription Group		Non-prescription Group		*P* Value	All		Pimobendan Prescription Group		Non-prescription Group		*P* Value
**n, %**		10 258	100.0%	473	100.0%	9,785	100.0%	-	552	100.0%	276	100.0%	276	100.0%	-
**Age**	Mean, SD	76.4	11.4	74.4	11.4	76.5	11.4	< 0.001	74.3	11.1	74.3	10.9	74.4	11.3	0.908
**Male**	n, %	5781	56.4%	323	68.3%	5,458	55.8%	< 0.001	367	66.5%	180	65.2%	187	67.8%	0.528
**Interval between hospitalizations for heart failure**	Median, (first quartile, third quartile)	162 (52, 397)		127 (41, 331)		164 (53, 400)		0.002	136 (39.5, 324.5)		119.5 (37.5, 292.5)		154.5 (43, 352.5)		0.069
**ADL score at discharge**	Mean, SD	19.0	1.9	19.2	1.7	18.9	1.9	0.003	19.4	1.6	19.4	1.5	19.4	1.6	0.978
**NYHA class**															
1	n, %	510	5.0%	13	2.7%	497	5.1%	0.021	18	3.3%	10	3.6%	8	2.9%	0.859
2	n, %	1941	18.9%	88	18.6%	1853	18.9%		164	29.7%	78	28.3%	86	31.2%	
3	n, %	2498	24.4%	140	29.6%	2358	24.1%		242	43.8%	124	44.9%	118	42.8%	
4	n, %	1776	17.3%	81	17.1%	1695	17.3%		128	23.2%	64	23.2%	64	23.2%	
Unclear/missing	n, %	3533	34.4%	151	31.9%	3382	34.6%		0	0.0%	0	0.0%	0	0.0%	
**Underlying heart disease**															
Ischemic heart disease	n, %	6839	66.7%	363	76.7%	6476	66.2%	<0.001	433	78.4%	216	78.3%	217	78.6%	0.918
Cardiomyopathy	n, %	1141	11.1%	118	24.9%	1023	10.5%	<0.001	136	24.6%	70	25.4%	66	23.9%	0.693
Valvular disease	n, %	3768	36.7%	174	36.8%	3594	36.7%	0.980	217	39.3%	108	39.1%	109	39.5%	0.931
Arrhythmia	n, %	3408	33.2%	212	44.8%	3196	32.7%	<0.001	245	44.4%	122	44.2%	123	44.6%	0.932
Other heart diseases	n, %	1741	17.0%	79	16.7%	1662	17.0%	0.873	94	17.0%	45	16.3%	49	17.8%	0.651
**Information on the hospital visit that served as the baseline for start of the follow-up period**															
Inpatient clinical department															
Cardiology department	n, %	5922	57.7%	289	61.1%	5633	57.6%	0.129	337	61.1%	163	59.1%	174	63.0%	0.337
Cardiovascular surgery	n, %	273	2.7%	7	1.5%	266	2.7%	0.102	8	1.4%	4	1.4%	4	1.4%	1.000
Others	n, %	4181	40.8%	183	38.7%	3998	40.9%	0.348	223	40.4%	114	41.3%	109	39.5%	0.665
Discharged to home	n, %	9813	95.7%	459	97.0%	9354	95.6%	0.132	537	97.3%	271	98.2%	266	96.4%	0.191
Duration (days) spent in hospital	Mean, SD	19.7	16.8	25.6	20.0	19.4	16.5	<0.001	24.6	19.7	23.9	16.7	25.3	22.2	0.417
**Concomitant drugs and therapy received during the hospital visit that served as the baseline for start of the follow-up period**															
Inotropic agents (intravenous injection)	n, %	1043	10.2%	146	30.9%	897	9.2%	<0.001	169	30.6%	88	31.9%	81	29.3%	0.518
Diuretics (intravenous injection)	n, %	6277	61.2%	291	61.5%	5986	61.2%	0.880	341	61.8%	169	61.2%	172	62.3%	0.793
Tolvaptan	n, %	2923	28.5%	221	46.7%	2702	27.6%	<0.001	238	43.1%	116	42.0%	122	44.2%	0.606
Carperitide	n, %	3614	35.2%	194	41.0%	3420	35.0%	0.007	265	48.0%	127	46.0%	138	50.0%	0.349
**Concomitant drugs and therapy during follow-up**															
ACE-I and/or ARB	n, %	6334	61.7%	293	61.9%	6041	61.7%	0.928	358	64.9%	175	63.4%	183	66.3%	0.476
β blocker	n, %	7014	68.4%	398	84.1%	6616	67.6%	<0.001	459	83.2%	231	83.7%	228	82.6%	0.733
Digitalis preparation	n, %	1000	9.7%	69	14.6%	931	9.5%	<0.001	83	15.0%	44	15.9%	39	14.1%	0.552
Diuretics (excluding tolvaptan and mineralocorticoid receptor antagonist)	n, %	9394	91.6%	461	97.5%	8933	91.3%	<0.001	541	98.0%	270	97.8%	271	98.2%	0.761
Tolvaptan	n, %	2672	26.0%	217	45.9%	2455	25.1%	<0.001	218	39.5%	114	41.3%	104	37.7%	0.384
Mineralocorticoid receptor antagonist	n, %	4831	47.1%	286	60.5%	4545	46.4%	<0.001	332	60.1%	170	61.6%	162	58.7%	0.487
Statin	n, %	4047	39.5%	203	42.9%	3844	39.3%	0.114	271	49.1%	123	44.6%	148	53.6%	0.033
Nitrates	n, %	2755	26.9%	144	30.4%	2611	26.7%	0.072	176	31.9%	86	31.2%	90	32.6%	0.715
Cardiac rehabilitation	n, %	702	6.8%	42	8.9%	660	6.7%	0.073	45	8.2%	24	8.7%	21	7.6%	0.641
**Concomitant disease during the hospital visit that served as the baseline for start of the follow-up period**															
Atrial fibrillation	n, %	5578	54.4%	277	58.6%	5301	54.2%	0.061	338	61.2%	167	60.5%	171	62.0%	0.727
Diabetes (including abnormal glucose tolerance)	n, %	5861	57.1%	308	65.1%	5553	56.8%	<0.001	354	64.1%	181	65.6%	173	62.7%	0.478
Hypertension	n, %	9288	90.5%	454	96.0%	8834	90.3%	<0.001	536	97.1%	265	96.0%	271	98.2%	0.128
Dyslipidemia	n, %	5723	55.8%	309	65.3%	5414	55.3%	<0.001	398	72.1%	190	68.8%	208	75.4%	0.088

Abbreviations: ACE-I, angiotensin-converting–enzyme inhibitor; ADL, activities of daily living; ARB, angiotensin II receptor blocker; CRT, cardiac resynchronization therapy; ICD, implantable cardioverter defibrillator; NYHA, New York Heart Association.

a For between-group comparison, the *t* test was used for continuous value data excluding interval between hospitalizations for heart failure and the chi-square test was used for categorical data.

Wilcoxon signed-rank test was used to compare interval between hospitalizations for heart failure.

**Table 2:** Frequency of Visits During Follow-Up^a^

		All		Pimobendan Prescription Group		Non-prescription Group		*P* Value
Frequency of visits during follow-up (days/month)	n	552		276		276		0.059

	Mean, SD	1.8	2.0	1.9	2.2	1.6	1.8	

	Median	1.3		1.3		1.2		

	Min, Max	0.0	17.6	0.4	17.6	0.0	14.1	

Duration of follow-up (months)	n	552		276		276		0.001
	Mean, SD	14.7	14.5	16.7	14.6	12.7	14.2	

	Median	9.3		10.8		7.5		
	Min, Max	0.3	75.4	0.5	74.4	0.3	75.4	

Abbreviations: Max, maximum value; Min, minimum value; SD, standard deviation.

a The frequency of visits was calculated from the duration of follow-up at evaluation of the primary endpoint using the following formula: frequency of visits = number of days visiting an outpatient clinic during follow-up/duration (months) after subtracting the duration of hospitalization from that of follow-up.

**Table 3:** Standardized Mean Difference of Patient Background at Evaluation of the Incidence of Rehospitalization for Heart Failure

	Pimobendan Prescription Group vs. Non-prescription Group (ref = Non-prescription Group)	
	Before Propensity Score Matching After Propensity Score Matching	
All, N	10 258	552
Pimobendan prescription group, n	473	276
Non-prescription group, n	9785	276
**Standardized mean difference (pimobendan prescription group-non-prescription group)**		
Age	0.18	0.01
Sex	−0.26	0.05
Interval between hospitalizations for heart failure	0.13	0.09
ADL score at discharge	−0.15	0.00
NYHA class	0.19	0.08
**Underlying heart disease**		
Ischemic heart disease	−0.24	0.01
Cardiomyopathy	−0.39	−0.03
Valvular disease	0.00	0.01
Arrhythmia	−0.25	0.01
Other heart diseases	0.01	0.04
**Information on hospital visit that served as the baseline for start of the follow-up period**		
Inpatient clinical department - Cardiology department	−0.07	0.08
Inpatient clinical department - Cardiovascular surgery	0.09	0.00
Inpatient clinical department–Others	0.04	−0.04
Discharge to home	−0.08	−0.11
Duration (days) spent in hospital	−0.34	0.07
**Concomitant drugs and therapy receive during the hospital visit that served as the baseline for start of the follow-up period**		
Inotropic agents (intravenous injection)	−0.56	−0.06
Diuretics (intravenous injection)	−0.01	0.02
Tolvaptan	−0.40	0.04
Carperitide	−0.13	0.08
**Concomitant drugs and therapy during follow-up**		
ACE-I and/or ARB	0.00	0.06
β blocker	−0.39	−0.03
Digitalis preparation	−0.16	−0.05
Diuretics (excluding tolvaptan and mineralocorticoid receptor antagonist)	−0.27	0.03
Tolvaptan	−0.45	−0.07
Mineralocorticoid receptor antagonist	−0.28	−0.06
Statin	−0.07	0.18
Nitrates	−0.08	0.03
Cardiac rehabilitation	−0.08	−0.04
**Concomitant disease during the hospital visit that served as the baseline for start of the follow-up period**		
Atrial fibrillation	−0.09	0.03
Diabetes (including abnormal glucose tolerance)	−0.17	−0.06
Hypertension	−0.23	0.13
Dyslipidemia	−0.21	0.15

Abbreviations: ACE-I, angiotensin-converting–enzyme inhibitor; ADL, activities of daily living; ARB, angiotensin II receptor blocker; CRT, cardiac resynchronization therapy; ICD, implantable cardioverter defibrillator; NYHA, New York Heart Association.

**Table 4:** Incidence of Rehospitalization for Heart Failure

	n	Rehospitalization	Number of People per Year Incidence		Incidence of Rehospitalization (/1000 People/Yr)			*P* Value (Chi-Square Test)
					95% Confidence Interval^a^			
Pimobendan prescription group	276	140	383.32	365.23	327.78	-	402.69	0.147
Non-prescription group	276	157	291.92	537.81	492.36	-	583.27	

a The 95% CI was calculated using the Poisson distribution.

**Table 5:** Annual Frequency of Rehospitalization for Heart Failure and Annual Frequency of All-Cause Hospitalizations

		Pimobendan Prescription Group		Non-prescription Group		*P* Value
Annual frequency of rehospitalization for heart failure	n	251		251		0.021
	Mean, SD	0.5	1.0	0.8	1.3	
	Median	0		0		
	Min, Max	0	6	0	9	
Annual frequency of all-cause hospitalizations	n	251		251		0.133
	Mean, SD	1.0	1.4	1.2	1.5	
	Median	1		1		
	Min, Max	0	8	0	9	

Abbreviations: Max, maximum value; Min, minimum value; SD, standard deviation.

**Table 6:** Incidence of Hospitalization for Adverse Events Characteristic to Pimobendan^a^

		n	Hospitalization for Adverse Events	Number of People per Year	Incidence of Rehospitalization (/1000 People/Yr)		*P* Value (Chi-Square Test)
					Incidence	95% Confidence Intervala	
Total	Pimobendan prescription group	251	3	468.31	6.41	1.45 – 11.37	0.315
	Non-prescription group	251	1	415.74	2.41	−0.63 – 5.45	
Torsade de pointes	Pimobendan prescription group	251	0	467.93	0.00	0.00 – 0.00	-
	Non-prescription group	251	0	415.74	0.00	0.00 – 0.00	
Transient ventricular fibrillation	Pimobendan prescription group	251	0	467.93	0.00	0.00 – 0.00	-
	Non-prescription group	251	0	415.74	0.00	0.00 – 0.00	
Ventricular fibrillation	Pimobendan prescription group	251	1	467.93	2.14	−0.73 – 5.00	0.317
	Non-prescription group	251	0	415.74	0.00	0.00 – 0.00	
Ventricular tachycardia	Pimobendan prescription group	251	1	467.93	2.14	−0.73 – 5.00	1.000
	Non-prescription group	251	1	415.74	2.41	−0.63 – 5.45	
Nonsustained ventricular tachycardia	Pimobendan prescription group	251	1	468.31	2.14	−0.73 – 5.00	0.317
	Non-prescription group	251	0	415.74	0.00	0.00 – 0.00	
Pulseless ventricular tachycardia	Pimobendan prescription group	251	0	467.93	0.00	0.00 – 0.00	-
	Non-prescription group	251	0	415.74	0.00	0.00 – 0.00	

a The 95% CI was calculated using the Poisson distribution.

**Table 7:** The Incidence of Rehospitalization for Heart Failure Stratified Based on the Interval of Index Hospitalizations^a^

Interval Between Hospitalizations for Heart Failure^a^		n	Rehospitalization	Number of People per Year	Incidence of Rehospitalization (/1000 People/Yr)		*P* Value (Chi-Square Test)
					Incidence	95% Confidence Interval^b^	
Shorter than 24 months	Pimobendan prescription group	258	135	359.97	375.03	337.08 – 412.99	0.215
	Non-prescription group	258	149	302.15	493.14	449.62 – 536.67	
Shorter than 12 months	Pimobendan prescription group	221	118	314.21	375.54	337.56 – 413.53	0.292
	Non-prescription group	221	129	274.28	470.32	427.81 – 512.82	
Shorter than 6 months	Pimobendan prescription group	168	84	234.68	357.94	320.86 – 395.02	0.189
	Non-prescription group	168	96	199.56	481.05	438.06 – 524.04	
Shorter than 3 months	Pimobendan prescription group	119	53	168.41	314.71	279.94 – 349.48	0.154
	Non-prescription group	119	64	108.70	588.75	541.20 – 636.31	

a The incidence of rehospitalization for heart failure in patient populations based on the interval between the first and second hospitalizations for heart failure, which served as the baseline of the follow-up start point: shorter than 24 months, shorter than 12 months, shorter than 6 months, and shorter than 3 months.

b The 95% CI was calculated using the Poisson distribution.
